# HIV, multimorbidity, and health-related quality of life in rural KwaZulu-Natal, South Africa: A population-based study

**DOI:** 10.1371/journal.pone.0293963

**Published:** 2024-02-21

**Authors:** Amelia M. Stanton, Ryan L. Boyd, Conall O’Cleirigh, Stephen Olivier, Brett Dolotina, Resign Gunda, Olivier Koole, Dickman Gareta, Tshwaraganang H. Modise, Zahra Reynolds, Thandeka Khoza, Kobus Herbst, Thumbi Ndung’u, Willem A. Hanekom, Emily B. Wong, Deenan Pillay, Mark J. Siedner, for the Vukuzazi Study Team

**Affiliations:** 1 Department of Psychological and Brain Sciences, Boston University, Boston, Massachusetts, United States of America; 2 Department of Psychiatry, Massachusetts General Hospital, Boston, Massachusetts, United States of America; 3 The Fenway Institute, Fenway Health, Boston, Massachusetts, United States of America; 4 The Obelus Institute, Washington, DC, United States of America; 5 Harvard Medical School, Boston, Massachusetts, United States of America; 6 Africa Health Research Institute, KwaZulu-Natal, South Africa; 7 Department of Epidemiology, Mailman School of Public Health, Columbia University, New York, New York, United States of America; 8 Division of Infection and Immunity, University College London, London, United Kingdom; 9 School of Nursing and Public Health, College of Health Sciences, University of KwaZulu-Natal, KwaZulu-Natal, South Africa; 10 Department of Clinical Research, London School of Hygiene and Tropical Medicine, London, United Kingdom; 11 Division of Infectious Diseases, Massachusetts General Hospital, Boston, Massachusetts, United States of America; 12 DSI-MRC South African Population Research Infrastructure Network (SAPRIN), South African Medical Research Council, Durban, South Africa; Iranian Institute for Health Sciences Research, ISLAMIC REPUBLIC OF IRAN

## Abstract

Health-related quality of life (HRQoL) assesses the perceived impact of health status across life domains. Although research has explored the relationship between specific conditions, including HIV, and HRQoL in low-resource settings, less attention has been paid to the association between multimorbidity and HRQoL. In a secondary analysis of cross-sectional data from the Vukuzazi (“Wake up and know ourselves” in isiZulu) study, which identified the prevalence and overlap of non-communicable and infectious diseases in the uMkhanyakunde district of KwaZulu-Natal, we (1) evaluated the impact of multimorbidity on HRQoL; (2) determined the relative associations among infectious diseases, non-communicable diseases (NCDs), and HRQoL; and (3) examined the effects of controlled versus non-controlled disease on HRQoL. HRQoL was measured using the EQ-5D-3L, which assesses overall perceived health, five specific domains (mobility, self-care, usual activities, pain/discomfort, and anxiety/depression), and three levels of problems (no problems, some problems, and extreme problems). Six diseases and disease states were included in this analysis: HIV, diabetes, stroke, heart attack, high blood pressure, and TB. After examining the degree to which number of conditions affects HRQoL, we estimated the effect of joint associations among combinations of diseases, each HRQoL domain, and overall health. Then, in one set of ridge regression models, we assessed the relative impact of HIV, diabetes, stroke, heart attack, high blood pressure, and tuberculosis on the HRQoL domains; in a second set of models, the contribution of treatment (controlled vs. uncontrolled disease) was added. A total of 14,008 individuals were included in this analysis. Having more conditions adversely affected perceived health (r = -0.060, p<0.001, 95% CI: -0.073 to -0.046) and all HRQoL domains. Infectious conditions were related to better perceived health (r = 0.051, p<0.001, 95% CI: 0.037 to 0.064) and better HRQoL, whereas non-communicable diseases (NCDs) were associated with worse perceived health (r = -0.124, p<0.001, -95% CI: 0.137 to -0.110) and lower HRQoL. Particular combinations of NCDs were detrimental to perceived health, whereas HIV, which was characterized by access to care and suppressed viral load in the large majority of those affected, was counterintuitively associated with better perceived health. With respect to disease control, unique combinations of uncontrolled NCDs were significantly related to worse perceived health, and controlled HIV was associated with better perceived health. The presence of controlled and uncontrolled NCDs was associated with poor perceived health and worse HRQoL, whereas the presence of controlled HIV was associated with improved HRQoL. HIV disease control may be critical for HRQoL among people with HIV, and incorporating NCD prevention and attention to multimorbidity into healthcare strategies may improve HRQoL.

## Introduction

Chronic diseases relapse and remit over time, with substantial impact on quality of life and daily functioning. Health-related quality of life (HRQoL) is a multidimensional construct that captures the perceived effects of illness and chronic disease, as well as associated treatments, on physical, emotional, and social wellbeing [1]. Among those living with health conditions for which efficacious treatments are available, long-term management and quality of life, rather than survival, are the ultimate goals. Yet, in sub-Saharan Africa and other low resource regions, people living with chronic illness—including HIV [2], tuberculosis (TB) [3], and diabetes [4]—report poor HRQoL, which has been associated with low adherence to medications [5] and other behaviors that negatively affect both physical and mental health [6, 7].

Although research has assessed the degree to which individual diseases and disease states impact HRQoL, less is known about the effects of multimorbidity—defined as multiple medical conditions occurring simultaneously in a single individual [8]—on HRQoL in sub-Saharan Africa (SSA). Data from high-income settings has isolated specific co-occurring disease groups or patterns of multimorbidity (e.g., cardiovascular and metabolic disease, mental health conditions, musculoskeletal disorders [9]) and demonstrated that patients with multimorbidity have increased utilization of healthcare services, reduced quality of life, and poorer health outcomes [10]. However, patterns of multimorbidity in high-income countries differ from those in low- and middle-income countries (LMICs), many of which are located in SSA. Countries in SSA have under-resourced health systems and similar disease profiles that characterized by emerging and re-emerging infectious and communicable diseases, included HIV, TB, and malaria. A recent epidemiological shift from infectious diseases to multiple non-communicable diseases (NCDs) signals a higher risk for mortality and disease-related disability in SSA than in other areas over the coming decades [11–13]. This shift has taken place in the context of large declines in HIV-related deaths and corresponding increases in life expectancy [14], with the HIV epidemic now concentrated in middle-aged groups that have high NCD risk. For example, in Uganda, the HIV prevalence rate had reduced to 6% by 2017, but rates among men and women aged 45–49 were much higher (14% and 11%, respectively) [15]. In South Africa, HIV prevalence rates are also highest among middle-aged men and women (approximately 26% and 38%, respectively) [16].

South Africa (SA), rapid demographic shifts to urban centers have contributed to changes in diet, decreases in physical activity, and increases in NCDs alongside established HIV and TB epidemics [17]. Moreover, whereas there has been tremendous investment and relative success in confronting the HIV, malaria, and TB epidemics in the region, less attention has been paid to NCD prevention and care [18]. Though a few recent studies have examined multimorbidity in SA [19–22], with a review estimating the prevalence of multimorbidity to be 3–23% in studies that included younger people and 30–87% in studies of older adults [23], none have addressed the impact of multimorbidity on HRQoL across a large cohort of community-dwelling adults with extensive phenotyping for both infectious diseases and NCDs. In addition, the effects of disease treatment (i.e., disease control) on HRQoL have yet to be examined in the context of multimorbidity.

The objectives of this secondary analysis of data collected for the Vukuzazi (“Wake up and know ourselves” in isiZulu) study [24] were to (1) evaluate the impact of multimorbidity on HRQoL; (2) determine the relative associations among infectious diseases, NCDs, and HRQoL; and (3) examine the effects of controlled versus non-controlled disease on HRQoL in a large South African cohort. This study may inform public health priorities and the design of interventions or services that target the needs of patients with multimorbidity, both in SA and in other countries that face a convergence of infectious and non-communicable disease epidemics.

## Methods

### Study setting and recruitment for the parent study

Adolescents and adults, aged 15 years and older, living in the Africa Health Research Institute (AHRI) Demographic Surveillance Area in the uMkhanyakunde district of KwaZulu-Natal were invited to participate in the parent study. Typical of rural SA, the uMkhanyakunde district has a high unemployment rate (58%), and one third of homes within the district do not have access to piped water.

Participants were recruited over an 18-month period, from May 2018 to November 2019, through home visits of all households in the catchment area. If no household members were home at the first visit, return visits were conducted. The recruitment team also issued proxy invitations to allow eligible household members who were away from the home at the time of the visit to participate in data collection. Approximately 50% of all adults in the area participated in the study. Please see Gunda et al. for a detailed cohort profile [25].

### Procedures of the parent study

All participants provided written informed consent. For non-emancipated participants under 18 years of age, we also obtained written parental consent for participation.

This was a secondary analysis of data collected for the Vukuzazi study [24], which leveraged an existing demographic and health surveillance cohort to determine the distribution and overlap of four common infectious and non-communicable diseases (HIV, TB, elevated blood glucose, and high blood pressure) in KwaZulu-Natal. The Vukuzazi data collection protocol is described briefly below (for a more detailed description, please see Wong et al., 2021) [24]. We used the STROBE cross-sectional reporting guidelines to complete this report [26].

Between May 2018 and November 2019, data collection was conducted via mobile health camp, a mobile facility where data is gathered on-site, in real-time, throughout the study area. At the mobile health camps, research nurses assessed history of tuberculosis (TB), HIV, hypertension, and diabetes. Participants who were not pregnant were screened for TB via digital chest x-ray, which were read in real-time by computer-assisted image analysis or by an experienced central radiologist [27]. From participants reporting current TB symptoms and participants with abnormal lung fields, sputum was collected and tested for *Mycobacterium tuberculosis (Mtb)* by Xpert MTP/RIF Ultra test (Cepheid, Sunnyvale, USA) and liquid mycobacterial culture (BACTEC MGIT 960 System, Becton Dickinson, Berkshire, UK). Blood was collected to measure glycosylated hemoglobin (HbA1c, VARIANT II TURBO Haemoglobin testing system; Bio-Rad, Marnes-la-Coquette, France) and to assess for HIV. Participants with a positive HIV immunoassay completed HIV-1 RNA viral load testing (Abbott RealTime HIV-1 Viral Load, Abbott, Illinois, USA).

After data collection, specimens were processed in the central laboratory, and results were reported back to participants. Normal results were communicated via text message. Abnormal results, including a new HIV diagnosis or a positive TB Xpert test, were reported by study nurses through home visits. These results were not available on the day that the EQ-5D-3L was administered; therefore, EQ-5D-3L results pertain to participants’ perceived HRQoL prior to receiving any information about new diagnoses.

### Data measurement for the current analyses

#### The predictor variables: Disease and disease control

Six diseases and disease states were included as predictor variables in this analysis: HIV, diabetes, stroke, heart attack, high blood pressure, and TB. Medical history and study assessments were used to (1) define the presence of the diseases and disease states included in the analysis and to (2) determine whether the disease was “controlled” (i.e., optimally managed via medical intervention).

Participants with a positive HIV immunoassay were defined as having HIV, and HIV disease was considered to be controlled if the following conditions were met: (1) HIV-1 RNA viral load < 40 copies/mL and (2) currently on antiretroviral therapy. Patients who were currently on antiretroviral therapy but did not have suppressed HIV-1 RNA viral loads were categorized as in treatment, with uncontrolled HIV. Diabetes was defined as hemoglobin A1c > 6.5% or self-reported use of diabetes medications in the past two weeks, irrespective of hemoglobin A1c. Controlled diabetes was defined per WHO’s STEPwise approach to NCD risk factor surveillance as self-reported use of diabetes management medications in the past two weeks with a hemoglobin A1c < 6.5% [28]. A lifetime history of stroke or heart attack was determined by self-report. High blood pressure was defined as a mean systolic BP ≥ 140 mmHg and diastolic BP ≥ 90 mmHg or a reported diagnosis of hypertension with use of anti-hypertensive medicines in the past two weeks. Finally, participants who were categorized as having active TB were either in the active phase of TB treatment at the time of the survey or had sputum that was positive for *Mtb* by Xpert MTB/RIF Ultra or liquid mycobacterial culture. Controlled TB was defined as currently receiving treatment for TB; participants who were categorized as having controlled TB were diagnosed with TB prior to completing the survey and were currently receiving treatment—at any phase—outside of the context of the study.

#### The outcome variable: HRQoL

HRQoLwas measured using the EQ-5D-3L, a well-known and widely used health status tool that was developed by the EuroQol Group [29]. The EQ-5D-3L includes a short descriptive system questionnaire that assesses five dimensions—mobility, self-care, usual activities, pain/discomfort, and anxiety/depression—and three levels of perceived problems (no problems, some problems, and extreme problems. The three levels are measured on a 3-point scale, which was recoded for ease of interpretation (with 3 indicating no problems, 2 indicating some problems, and 1 indicating extreme problems), creating a descriptive profile of a respondent’s health state. This descriptive system is linked to a value set (i.e., a set of weights for each health state description that varies by country/region), but value sets were not used in the current study. The measure also includes a visual analog scale (EQ VAS) that assesses overall health (“We would like to know how good or bad your health is TODAY”) on a scale from 0 to 100, with 0 defined as “the worst health you can imagine” and 100 defined as “the best health you can imagine”. The EQ-5D-3L was chosen over other widely-used tools (e.g., SF-36 [30], SF-12 [31–33]) first and foremost for its brevity, which was essential for use in a large sample, but also for its validation across languages (over 150, including multiple South African languages) and reproducibility.

### Statistical analyses

Two broad analytic strategies were used to understand multimorbidity’s impact on HRQoL. First, Pearson correlational analyses were conducted to understand the general relationships among participants’ number of diagnosed conditions (noninfectious, infectious, and total), overall perceived health, and the five HRQoL domains. Second, to understand the complex inter-relationships among all disease states, level of disease control, and their cumulative impact on HRQoL, we employed a regularized regression strategy known as “elastic net”, a process of selecting predictor variables for multiple regression through a sophisticated penalization strategy or shrinking algorithm. The elastic net process is flexible and data-driven, such that the statistics determine the precise parameters of the shrinking algorithm that is applied. A standard ridge regression approach, which implicitly accounts for the high degree of intercorrelations among the variables, ultimately emerged from the data [34]. This approach statistically manages and overcomes issues of multicollinearity, which may have compromised the results if left unaddressed (variance inflation factors are reported in S1 Table). Ridge regression is not only recommended for its ability to handle intercorrelations among predictors but, critically, is valuable for its ability to minimize overfitting [35]. Importantly, the ridge regressions enabled us to prioritize our goal of understanding of the roles that all conditions play in HRQoL rather than simpler predictor combinations–specifically, in a more accurate manner than afforded by other traditional regression approaches [36, 37].

We employed *k*-fold cross-validation procedures during the modeling pipeline at several stages, including the parameter optimization and model evaluation processes, to prevent arbitrary overfitting of the data and to compare models against each other. The shrinkage parameter was tuned via the cva.glmnet function of the glmnetUtils package in R [38]. This function is designed to tune the shrinkage parameter via cross-validation. Specifically, we used cross-validation to identify optimal elastic net parameters for our initial modeling of multimorbidity’s impact on HRQoL domains [39]. Then, we performed additional 10-fold cross-validation of our ridge regressions to derive conservative, realistic estimates of our account of variance so as to not report R-squared values from overfit models.

We controlled for age and gender because they are generally considered to be confounding variables [40, 41]; between-group comparisons (see Table 1) and Pearson correlations (see Supporting Information) also suggested that age and gender were potential confounds in our sample, which provided further justification for residualizing age and gender out of the models described below. Even though HRQoL was measured on an ordinal scale, we did not use ordinal regressions because modeling out the confounds of age and gender does not produce ordinal residuals; therefore, per established guidelines [42, 43], we treated the residuals as continuous variables for the primary analyses.

**Table 1:** Demographics, disease states, total number of disease conditions, and HRQoL domains by gender.

	Women	Men	t-value/z-value^†^
Age (M, SD)	41.72 (18.88)	35.88 (18.52)	17.15***
Disease states (n, %)–*Predictor variables for subsequent models*			
HIV	3850 (40.20)	1139 (25.70)	16.57***
Diabetes	1130 (11.80)	239 (5.39)	11.60***
Stroke	224 (2.33)	46 (1.04)	5.076***
Heart attack	115 (1.20)	23 (0.52)	3.696***
High blood pressure	2565 (26.80)	709 (16.00)	13.91***
Active TB	163 (0.02)	191 (0.04)	-8.823***
Number of conditions (M, SD)	0.84 (0.79)	0.52 (0.72)	22.27***
Number of infectious conditions (M, SD)	0.42 (0.30)	0.51 (0.50)	12.90***
Number of non-infectious conditions (M, SD)	0.42 (0.68)	0.23 (0.50)	16.87***
Quality of life domains (M, SD)^a^ –*Outcome variables for subsequent models*			
Overall health (EQ VAS)	87.43 (15.17)	90.70 (13.31)	-12.23***
Mobility	2.86 (0.34)	2.92 (0.27)	-9.542***
Pain/discomfort	2.76 (0.46)	2.86 (0.36)	-12.84***
Self-Care	2.88 (0.33)	2.93 (0.34)	-8.771***
Usual activity	2.82 (0.39)	2.89 (0.32)	-10.37***
Anxiety/Depression	2.85 (0.37)	2.91 (0.30)	-8.368***

*** p < 0.001

^†^ Continuous measures (age, numbers of conditions, and quality of life domains) were compared via an independent-samples t-test (t-values are reported). Categorical measures (i.e., positive diagnosis of disease states) compared via binomial regression (z-values are reported).

^a^Note that the quality of life domains were assessed on a three-point scale (1–3), which were recoded for ease of interpretation so that 1 indicated extreme problems and 3 indicated no problems.

We ran two sets of regression models. In the first set of models, we examined each disease and disease state as predictors of overall health and each of the five HRQoL domains. In the second set, we included variables reflecting disease control to assess the degree to which treatment influenced the relationships among comorbid disease and the HRQoL domains. All analyses were conducted in the R software environment using the glmnet package [44, 45], with significance test estimates generated using Cule et al.’s method [46]. We note, however, that such estimates for penalized regressions are problematic and, while heuristically useful, we strongly caution against overinterpretation (see [47, 48]). Finally, we ran follow-up relative weights analyses to test for whether some conditions played significantly stronger roles in HRQoL than others within any given model.

### Patient and public involvement

The AHRI Community Advisory Board contributed to the study design and the selection of the measures. The Community Advisory Board also reviewed and approved the study protocol. Throughout each phase of the project, the AHRI Public Engagement Department routinely shared study results via public communications and road shows.

## Results

### Study participation and demographics

Out of the 18,053 participants who enrolled in the Vukuzazi study, 14,008 individuals were included in this analysis (4,045 participants were excluded because they enrolled prior to inclusion of the EQ5D HRQoL questionnaire). Women comprised the majority of the final sub-sample (N = 9,573; 68.34%), and the average age of all participants was 39.87 years (SD = 18.96). See Table 1 for information on demographics, the prevalence of each of the six diseases and disease states, and the HRQoL domains by gender.

With respect to HRQoL, most participants (80–90%) reported no problems with mobility, pain/discomfort, self-care, usual activity, and anxiety/depression, 10–20% of participants reported some problems across the five domains, and less than 2% of participants reported extreme problems across the five domains (Table 2).

**Table 2:** EQ-5D-3L scores across the five domains.

	Mobility	Pain/Discomfort	Self-Care	Usual Activity	Anxiety/ Depression
No Problems	12369 (88.30%)	11297 (80.65%)	12532 (89.46%)	11873 (84.76%)	12298 (87.79%)
Some Problems	1630 (11.64%)	2539 (18.13%)	1448 (10.34%)	2078 (14.83%)	1644 (11.74%)
Extreme problems	9 (00.06%)	172 (01.23%)	28 (00.20%)	57 (00.41%)	66 (00.47%)

The prevalence rates of multimorbidity were as follows: 14% of participants had two conditions, 2% of participants had three conditions, 0.24% had four conditions, and 0.007% had five conditions (Table 3). We also provide a breakdown of perceived overall health by number of conditions, number of non-infectious, and number of infectious conditions in Table 3.

**Table 3:** Prevalence of multimorbidity and perceived overall health by number of conditions, number of non-infectious conditions, and number of infectious conditions, controlling for age and gender.

	N (%)	Mean of Overall Health	Standard deviation	Median of Overall Health
Number of conditions (Fig 1)				
0	6600 (47.12)	74.73	9.54	77.02
1	6011 (42.91)	75.30**	10.69	77.52
2	1965 (14.03)	73.39***	12.43	74.89
3	329 (2.35)	70.69***	14.27	72.73
4	33 (0.24)	68.23	11.76	67.22
5	1 (0.007)	69.70	NA	69.70
Number of non-infectious conditions (Fig 2)				
0	10660 (76.1)	75.20	9.60	77.20
1	3160 (22.56)	74.04***	12.08	75.33
2	1021 (7.29)	71.81***	13.82	73.47
3	92 (0.66)	66.50***	13.38	67.12
4	6 (0.04)	71.32	5.17	69.20
Number of infectious conditions (Fig 3)				
0	9568 (68.3)	74.26	10.70	76.82
1	5179 (36.97)	75.41***	10.20	78.01
2	192 (1.37)	74.84	11.68	77.52

** *p* < 0.01

*** p < 0.001

These p-values correspond to statistically differences between X number of conditions and the preceding number of conditions (X-1). For example, the difference between the mean value of perceived overall health among participants with 3 total conditions (70.69) is significantly lower than the mean value of perceived overall health among participants with 2 total conditions at the p <0.001 level.

### Multimorbidity and health-related quality of life

Correlational analyses revealed that more conditions, regardless of condition type (non-communicable vs. infectious), was associated with poor perceived overall health (measured via the EQ VAS) and with decreased HRQoL across the five domains (see Table 3 for Pearson correlation coefficients). Similarly, correlational analyses demonstrated that higher numbers of comorbid NCDs were associated with worse overall health and decreased HRQoL across the five domains (see Table 4). The strongest negative correlations between number of NCDs and the HRQoL domains were for usual activity (*r* = -0.136) and mobility (*r* = -0.134). However, unlike the relationships among number of NCDs and the HRQoL domains, higher numbers of infectious diseases were positively associated with overall health and HRQoL. The strongest positive correlation was between number of infectious diseases and mobility (*r* = 0.097). With a sample size of 14,008, differences between coefficients (i.e., differences in the strengths of the relationships) of a magnitude ≥ 0.017 were associated with a p-value < 0.05 (see Table 4 note). For example, the correlation between overall perceived health and number of conditions (-0.060) is a significantly stronger association than the correlation between mobility and number of conditions (-0.032). Differences in the strength of these relationships were assessed using the cocor package in R [49].

**Table 4:** Pearson correlation coefficients among HRQoL domains, total number of conditions, number of infectious conditions, and number of non-infectious conditions, after controlling for age and gender.

Domain	Number of Conditions	Number of Infectious Conditions	Number of Non-Infectious Conditions
Overall Health (EQ VAS)	-0.060 [-0.073, -0.046]	0.051 [0.037, 0.064]	-0.124 [-0.137, -0.110]
Mobility	-0.032 [-0.045, -0.018]	0.097 [0.084, 0.110]	-0.134 [-0.147, -0.121]
Pain/Discomfort	-0.042 [-0.055, -0.028]	0.073 [0.060, 0.086]	-0.125 [-0.138, -0.112]
Self-Care	-0.025* [-0.038, -0.011]	0.082 [0.069, 0.095]	-0.111 [-0.125, -0.098]
Usual Activity	-0.045 [-0.059, -0.032]	0.080 [0.066, 0.093]	-0.136 [-0.149, -0.123]
Anxiety/ Depression	-0.048 [-0.062, -0.035]	0.041 [0.028, 0.054]	-0.102 [-0.115, -0.089]

* p = 0.002; all other *p*s < 0.001.

Note. Given our sample size and the generally high intercorrelations between HRQoL measures (Cronbach’s α = 0.86) and number of conditions (α = 0.69), differences between coefficients of a magnitude ≥ 0.017 were associated with a p-value < 0.05.

In the first set of regression models, combinations of NCDs were associated with lower overall health and lower HRQoL for each of the domains. Table 5 presents the relative contribution of each disease to each HRQoL domain. For any given disease/predictor, the beta values reflect the change in each HRQoL domain, measured by a tool that has been rescaled and residualized (per the description in the Methods), as a function of the presence of a given condition or disease state.

**Table 5:** Beta weights for diseases/disease states that were significantly associated with overall health and/or the five HRQoL domains in the first set of regression models (Model 1).

Predictor	Overall Health	Mobility	Pain/ Discomfort	Self-Care	Usual Activity	Anxiety/ Depression
Intercept	74.984	4.135	3.818	4.177	3.965	4.126
Diabetes	-0.156*	-0.006^†^	-0.015***	-0.008*	-0.011**	-0.016***
History of stroke	-0.932***	-0.039***	-0.045***	-0.031***	-0.038***	-0.040***
History of heart attack	-0.339***	-0.008*	-0.023***	-0.005	-0.013***	-0.013**
HIV	0.354***	0.036***	0.032***	0.034***	0.031***	0.019***
Hypertension	-0.918***	-0.040***	-0.036***	-0.033***	-0.045***	-0.023***
Active TB	-0.020***	0.001	0.001	-0.006^†^	-0.001	-0.005
*R* ^2^	0.023	0.029	0.024	0.021	0.027	0.016
10-fold cross-validation *R*^2^	0.023	0.029	0.024	0.020	0.027	0.016

^†^ *p* < 0.10

* p < 0.05

** *p* < 0.01

*** p < 0.001

The majority of diagnosed conditions contributed negatively to perceived overall health. Notably, a history of stroke and current hypertension were most strongly associated with poorer overall health (β = -0.932 and β = -0.918, respectively). HIV was most strongly associated with better overall health (β = 0.354). It is important to note that this result is counterintuitive; we address factors that may be driving this association, including the role of treatment and disease control, in the results section below and in the Discussion. These relationships were fairly consistent across the five HRQoL domains (see Table 5). Combining the beta weights for individual diseases or disease states provides cumulative estimates of the strength of the association between a set of comorbid or multimorbid conditions and each of the HRQoL domains (i.e., beta weights are additive). For example, while stroke and hypertension each detract from HRQoL, perceived overall health is negatively impacted if a person is diagnosed with both relative to either condition individually. For a quantification of multicollinearity in this model, please see the Supporting Information, which include a variance inflation factor (VIF) table. Any VIF above 1 indicates a modest degree of collinearity.

**Fig 1:**
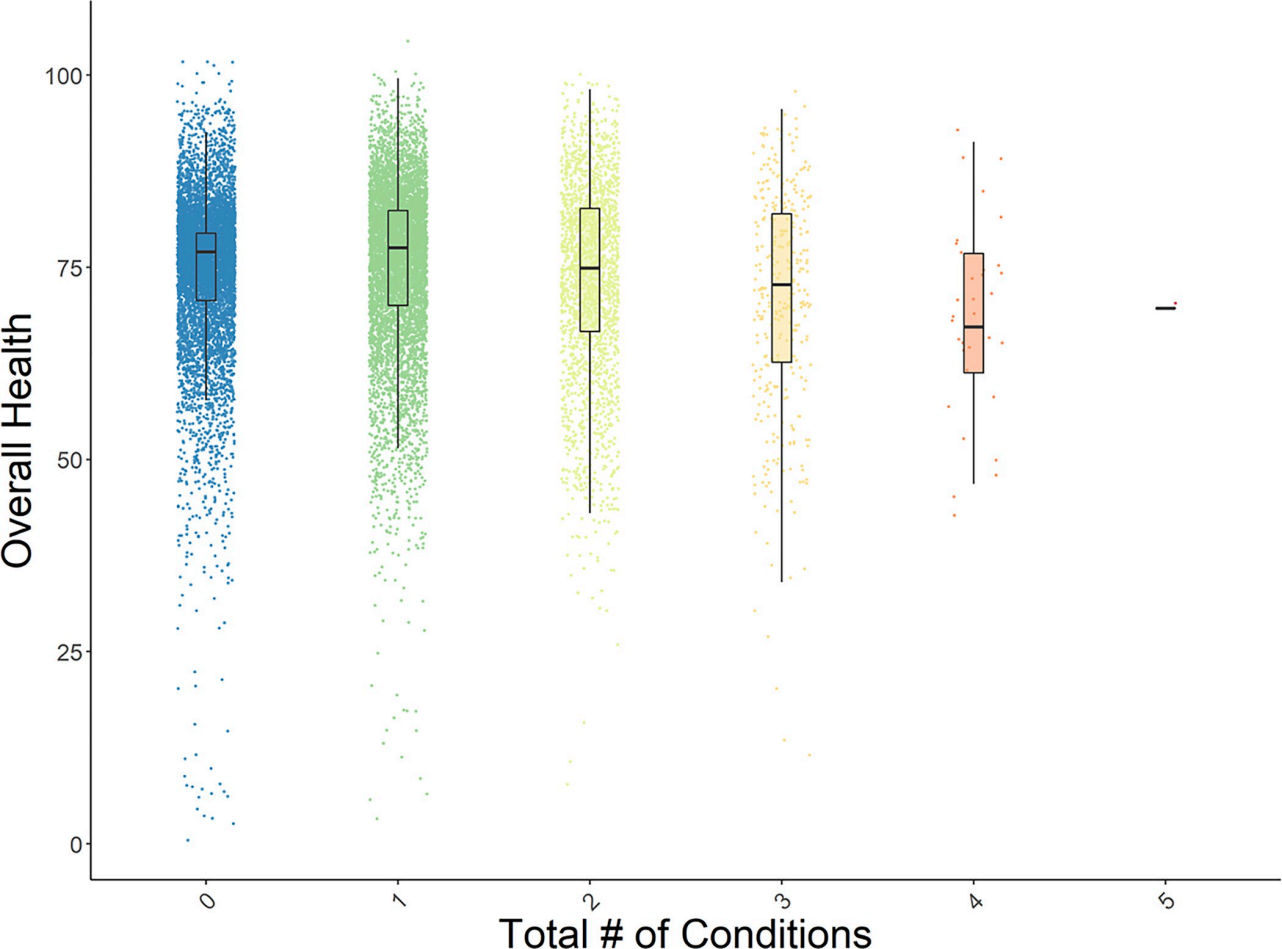
Box plot of the distribution of perceived overall health by number of conditions, with age and gender residualized. The plots present the medians of the data.

**Fig 2:**
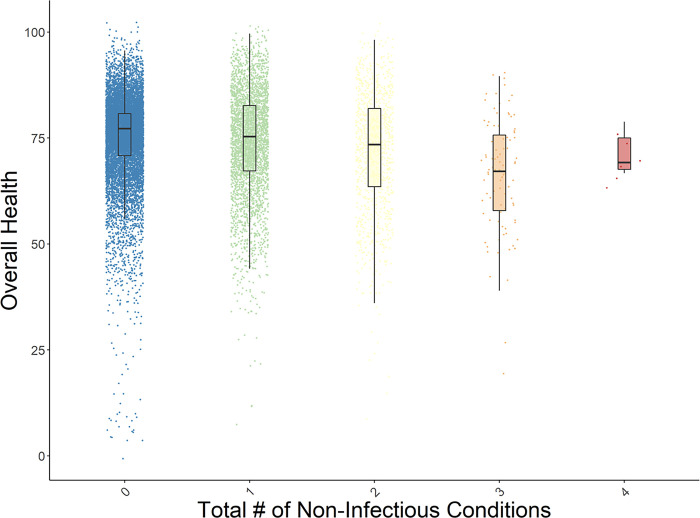
Box plot of the distribution of perceived overall health by number of non-infectious conditions, with age and gender residualized. The plots present the medians of the data.

**Fig 3:**
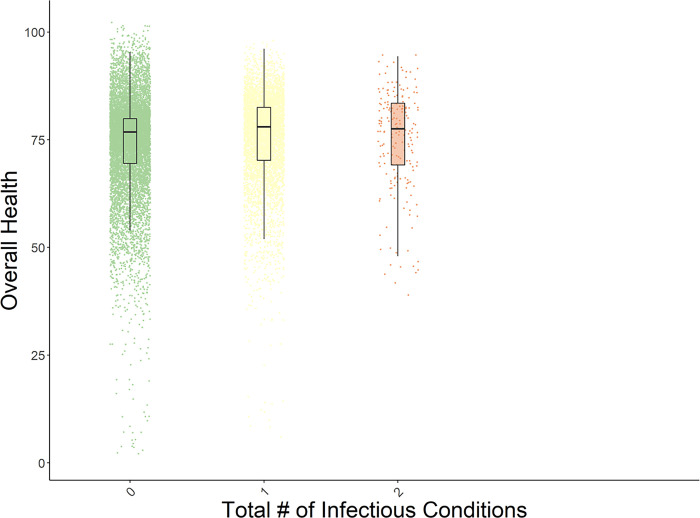
Box plot of the distribution of perceived overall health by number of infectious conditions, with age and gender residualized. The plots present the medians of the data.

See Figs 1–3 for box plots of the associations between number of conditions and perceived health, number of NCDs and perceived health, and number of infectious diseases and perceived health. Each figure indicates the medians for overall health ratings per number of conditions, controlling for age and gender. Similar relationships were evident across all five HRQoL domains. These box plots depict the relative decreases in perceived health as multimorbidity increases. Table 3 presents the descriptive data (means, standard deviations, and medians) that are associated with the Figures.

### The role of treatment in the relationship between multimorbidity and health-related quality of life

To understand the impact of disease treatment on HRQoL, a second set of regression models were performed parallel to those described above, albeit with more nuanced measures of diseases ranging from diagnosed but uncontrolled to diagnosed and medically controlled; Table 5 presents results from these analyses. Much like the first set of models, uncontrolled hypertension was most strongly associated with poor health (β = -0.918), as was history of stroke (β = -0.913), and; similarly, most diagnosed disease and disease states, whether controlled or uncontrolled, negatively impacted HRQoL. The strength of the associations among HIV, overall health, and the HRQoL domains differed based on level of disease control; that is, the relationship between controlled HIV and good overall health was stronger than the relationship between uncontrolled HIV and good health (β = 0.383 vs. β = 0.145, respectively). In a follow-up bootstrapped relative weights analysis, the difference between these two beta weights had a p-value < .05, CI = [-0.0032, -0.0003] (see [50]). Again, combining the beta weights for individual diseases or disease states provides an estimate of the strength of the association between a set of comorbid/multimorbid conditions and each of the HRQoL domains. For a quantification of multicollinearity in this model, please see the VIF table in the Supporting Information.

Finally, using a five-by-two-fold cross validation procedure as described in the Methods, we compared the set of models that accounts for treatment/disease control with the set of models that do not; R-squared values were compared to each other using a paired samples t-test (see Table 6) [51]. In the models estimating overall perceived health, the model that included disease control variables accounted for significantly more variance than the model that did not include those variables (5×2 paired-*t*(9)_Δ*R*_^2^ = 11.116, *p* < 0.001). With respect to the anxiety/depression domain of HRQoL, the model with disease control variables accounted for significantly less variance than the model without those variables (5×2 paired-*t*(9)_Δ*R*_^2^ = -3.084, *p* < 0.01). However, the differences in the expected R-squared values derived from cross-validation suggest that, in general, the models are functionally comparable in their ability to account for variance. Therefore, in the case of the anxiety/depression models, one should not necessarily be seen as “better” or “worse” from a predictive standpoint, as these differences in variance accounted for are so small as to be arbitrary.

**Table 6:** Beta weights for diseases/disease states that were significantly associated with overall health and/or the five HRQoL domains in the second set of regression models (Model 2), which included variables that indicate disease control.

Predictor	Overall Health	Mobility	Pain/ Discomfort	Self-Care	Usual Activity	Anxiety/ Depression
Intercept	74.984	4.135	3.818	4.177	3.965	4.126
Current smoker	-0.504***	0.003	-0.003	0.009	0.002	-0.002
Controlled diabetes	-0.076	-0.010**	-0.008^†^	-0.010**	-0.013***	-0.009*
Uncontrolled diabetes	-0.161**	-0.004	-0.014**	-0.006	-0.008*	-0.014***
History of stroke	-0.913***	-0.039***	-0.044***	-0.030***	-0.037***	-0.039***
History of heart attack	-0.326***	-0.008*	-0.022***	-0.005	-0.013***	-0.012**
Controlled HIV	0.383***	0.035***	0.029***	0.032***	0.029***	0.020***
On treatment, but uncontrolled HIV	0.058	0.009*	0.012**	0.008*	0.010**	0.005
Uncontrolled HIV	0.145	0.014***	0.015***	0.013***	0.013***	0.003
Hypertension, uncontrolled	-0.918***	-0.040***	-0.036***	-0.032***	-0.044***	-0.022***
Active TB	0.024	0.001	0.001	-0.005*	0.000	-0.004
Controlled TB	-0.314***	-0.002	-0.007^†^	-0.007*	-0.006	-0.007*
*R* ^2^	0.027	0.029	0.025	0.022	0.027	.017
10-fold cross-validation *R*^2^	0.026	0.028	0.024	0.020	0.027	.015
5×2 paired-*t*(9)_Δ*R*_^2^	11.116***	-0.683	-1.606	0.717	-1.011	-3.084**

^†^ *p* < 0.10

* p < 0.05

** *p* < 0.01

*** p < 0.001

## Discussion

In a large sample of adults living in rural KwaZulu-Natal, multimorbidity was associated with poor HRQoL; as number of conditions increased, HRQoL decreased across all domains. Notably, the impact of multimorbidity on HRQoL differed by type and management of disease. The presence of both controlled and uncontrolled NCDs was associated with poorer HRQoL, whereas the presence of controlled infectious diseases, driven by high rates of disease control among people with HIV, was associated with higher HRQoL. Unlike most previous studies, which either determined the prevalence of multimorbidity in certain regions or assessed the impact of specific diseases on HRQoL, this analysis was the first to demonstrate the association of treatment on the relationship between multimorbidity and HRQoL. Understanding the degree to which disease control is related to overall functioning has important implications for health policy and health service delivery in low-resource settings, as it may enable treatment prioritization based on individual patient values and risk for poor HRQoL.

Data from high-resource settings consistently demonstrate that HRQoL decreases as number of conditions increases. A recent meta-analysis indicated that the mean decrease in HRQoL per each added disease ranged from 1.55% to 4.37%, depending on the HRQoL scale that was used [52]. The majority of the studies included in the meta-analysis were performed in high-income countries, with only one SA-based study representing the entire African continent. Although the relationship between increasing number of diseases and poor HRQoL may seem evident, the nuances of this relationship likely differ between high-income settings, which have lower prevalence rates of infectious diseases, and low-income or middle-income settings, where NCD and infectious disease epidemics co-occur.

When the contribution of multimorbidity to HRQoL was examined by disease type, the impact of NCDs was negative, as was the impact of TB. An HIV diagnosis, however, was associated with better HRQoL, which was counterintuitive, given that the negative influence of HIV-related symptoms on both physical and mental HRQoL domains has been well-documented [53, 54]. However, the role of HIV treatment is meaningful here. Successful engagement in care, adherence to therapy and virologic suppression is often associated with a return to health, sometimes after grave illness, and significant improvements in HRQoL [55]. HIV care is also associated with increased social support and other pro-health behaviors, thereby potentially increasing HRQoL. Indeed, when disease control was factored into the models, controlled HIV was associated with greater HRQoL than uncontrolled HIV, suggesting a possible positive impact of HIV treatment on HRQoL. Similar effects of antiretroviral therapy (ART) on HRQoL among people with HIV have been previously reported in both South Africa and Zambia [56]. Notably, this population-based cohort included individuals with relatively well controlled HIV; 83% of participants living with HIV were virologically suppressed, with a median CD4 count of 693 cells/uL (SD = 340.40). These data add to growing evidence for positive secondary effects of HIV care that are not always available those without HIV infection [57].

With the possibility of these secondary effects in mind, it is important to consider the societal and health systems implications of the association between well-controlled HIV and better perceived health across domains, as well as the implications of the stronger association between controlled HIV and better perceived health. A potential hypothesis that would explain this counterintuitive phenomenon is that HIV care provides greater frequency and intensity of contact between individuals and their health care providers, counselors, pharmacists, and the health care system than what is typically received for those without a chronic disease (or those with one, such as hypertension and diabetes, which is not as well addressed by the South African healthcare system). This increased interface with healthcare providers may in turn increase opportunities for health education as well as improve health literacy, health screening and maintenance, and other such services, which may ultimately have downstream effects on different domains of HRQoL. For example, numerous studies have demonstrated that people with HIV in sub-Saharan Africa have improved health indicators for other conditions, such as diabetes, hypertension, and atherosclerosis, and that HIV care may lead to increases in smoking cessation [58–63]. Though cost effectiveness will need to be considered, the integrated ART program model may be harnessed to increase touchpoints for care in populations with NCDs or comorbid HIV/NCDs [64], especially if longitudinal studies support the association between controlled HIV and better perceived HRQoL and if mediational analyses suggest that strong relationships with providers and associated social support may be driving these relationships.

Other studies have also found that ART is associated with improved HRQoL in sub-Saharan contexts [65, 66], but few have done so in a sample of this size, and none have examined these relationships in the context of comorbidities. Although treatment engagement lessened the negative effects of some of these disease states on HRQoL, controlled NCDs either had no significant impact on HRQoL or were associated with poor HRQoL. This suggests that, in SA, public health efforts that aim to prevent NCDs may have a meaningful impact on HRQoL. These efforts could be integrated into or independent of HIV treatment.

It is important to address the implications of the size of the correlation coefficients and the beta weights that we report in this analysis. Although the coefficients and beta weights that we report are small, they are typical of studies that use self-report measures to assess psychological constructs. In fact, the beta weights that we report are actually larger than others documented in the multimorbidity literature [67]. Even models that include variables like age and sex may at best account for 14% of the total variance, for example [68], corresponding to an overall correlation of ~.37. There is also a substantial established literature on the interpretation of effect sizes, particularly those reported in real-world contexts (i.e., outside of the lab). These papers typically stress the importance of small effects, especially when they occur in non-trivial contexts, challenge existing theory and assumptions, and may have large cumulative consequences if replicated [69, 70]. Many of our findings fit all three criteria and therefore warrant documentation and further exploration in future work. Furthermore, the statistically significant differences in correlation strengths provide important clinical insights; this nuanced information about which disease states and associated treatments may impact HRQoL could help inform treatment priorities and decision-making, especially in resource-limited settings, where some treatments may need to be prioritized over others.

With NCDs projected to account for nearly half of the burden of disease in low-income countries by 2030 [71], awareness of the relationships between these diseases and HRQoL reinforces the importance of early identification of modifiable risk factors in maintaining population health, functioning, and overall quality of life. In settings with high HIV-burden like SA, multimorbidity is occurring at younger ages, as younger individuals living with HIV are at higher risk for heart-related conditions [72] and have increased levels of cholesterol and triglycerides compared to uninfected adolescents [73]. Missed opportunities to address modifiable NCD risk factors, as early as adolescence [74], may strain under-resourced health systems that are not yet equipped to manage multimorbidity and have long-term negative effects, given that NCD disease treatment does not appear to be associated with improved HRQoL.

Several limitations of these data should be acknowledged. First, two thirds of the sample were women, which may have resulted from bias during data collection. Given this imbalance, we chose to residualize gender out of all variables. In addition, though the study used a full population and recruited individuals directly from the community to minimize healthcare seeking bias, approximately 50% of the eligible population enrolled. As such, there may be residual bias related to participation. As described by Gunda and colleagues [25], contact rates were low among participants who were unavailable due to work or other commitments, which made it difficult to enroll specific subpopulations (e.g., working men). Non-participation could therefore be non-random, which should be considered when interpreting disease prevalences and HRQoL and should be investigated in future studies. Second, the blood pressure and blood glucose measurements that were used to define hypertension and diabetes were captured on a single day. To confirm these diagnoses, additional measurements need to be captured over time. Moreover, some disease state measurements were self-reported, whereas others were directly measured. For example, self-reported histories of disease conditions (e.g., lifetime history of stroke, heart attack) were not confirmed by medical providers or medical records, which may have introduced bias. Third, several key categories of NCDs were not assessed in this study, including cancer and chronic respiratory disease, which may have unique associations with HRQoL or interactions with other comorbid conditions that may impact HRQoL. Logistical barriers and ethical considerations informed the selection of diseases that were assessed. Investigators considered the burden that expanded health screenings might place on rural primary health care providers and clinics, and in choosing to measure certain diseases, interagency and interdisciplinary collaboration was required to establish appropriate referral and treatment pathways. In addition, given the extensive nature of the project, other important and nuanced factors that may impact HRQoL—including but not limited to socioeconomic status, structural issues, social circumstances, interpersonal relationships, discrimination and other forms of stigma—are difficult to measure and were not assessed in this study, and therefore could not be included in the analyses. Similarly, other measurement instruments—for example, HRQoL tools that were developed specifically for people with HIV—would have been valuable, but as one might expect, the logistics of a study of this magnitude (total N > 18,000) required the incorporation of brief, reproducible, validated scales (such as the EQ-5D-3L), with a view to characterizing the entire population, independent of any single comorbidity (e.g., HIV). Fourth, we could not account for the timing or severity of diagnoses and associated treatments, which may impact HRQoL, nor did we account for the interpersonal, community, and structural factors that may have protected against the negative effects of diseases and their treatments on HRQoL. Notably, the EQ-5D-3L assessment was administered to participants before they received any information about new diagnoses. It is possible that knowledge of a new diagnosis or a change in health status may have influenced responding had the assessment been administered after participants received all of their final results. The relationship between knowledge of health status and HRQoL should be explored in future research. Fifth, the current data and analytic methods precluded the broadband inclusion of all possible interaction terms within our dataset, limiting the degree to which our model recognizes the ways in which one disease may amplify or suppress the impact of other diseases on HRQoL. That is, two conditions or disease states may interact to produce a worse outcome than the additive effect of both conditions combined. While we acknowledge that there are likely interactive effects between conditions that extend beyond a summative process, modeling all interaction terms would be neither feasible nor interpretable with the current sample. Finally, as with all cross-sectional data, these results do not speak to causal associations. To gain insight on causal relationships in the context of multimorbidity, future studies should conduct longitudinal examinations of HRQoL that start from prevention programming and continue throughout treatment trajectories. Future work should also thoroughly assess the structural and social determinants of HRQoL and include these variables in models, alongside those indicating different levels of disease control. The inclusion of those data would enable a holistic representation of the multidimensional factors that may be associated with HRQoL. Until these additional studies are conducted, these findings should be interpreted with caution.

Under ideal circumstances, multimorbidity would be addressed by providers who are trained to consider quality of life in the management of complex co-occurring conditions, and prevention programs would specifically target diseases and disease states that most negatively impact HRQoL. Emerging programs that address mental health in the context of comorbid HIV disease through stepped care [75] or task shifted approaches [76] may be an important model to support improved HRQoL among people living with HIV in Southern Africa. In SA, an integrated chronic disease model has been proposed and variably implemented [77], and pilot programs that facilitate integrated treatment for infectious diseases and NCDs are underway [78]. Some preliminary feedback on these programs from both providers and patients has highlighted structural and process-oriented implementation challenges [79], whereas other studies have found high levels of fidelity in implementing the integrated model [80] and high satisfaction with integrated services [81]. Although integration of services for comorbid conditions is critical, more attention must be paid to the development and implementation of interventions that aim to prevent stroke, heart attack, and other NCDs, particularly in populations that have elevated risk for NCDs, such as women living with HIV [82].

## Conclusion

In conclusion, we found that presence of NCDs, whether controlled or not, was associated with poor HRQoL. However, the presence of HIV was associated with higher HRQoL, and the strength of that relationship increased when HIV was controlled. These findings suggest that investing in programs that prevent NCDs will likely have a meaningful impact on HRQoL in SA and other contexts where NCD and infectious disease epidemics converge.

## Supporting information

S1 File List of Vukuzazi team members. (PDF)

S1 Table Pearson correlation coefficients for age/gender, disease states, and HRQoL domains. (DOCX)

S2 Table Variance inflation factors (VIF) for the first and second set of regression models. (DOCX)
