# Supplementary material for: HIV, multimorbidity, and health-related quality of life in rural KwaZulu-Natal, South Africa: A population-based study
Source: PLoS One. 2024 Feb 21;19(2):e0293963. doi: 10.1371/journal.pone.0293963 (PMC10880982; doi:10.1371/journal.pone.0293963)
Supplement: S1 Table — (DOCX) [file pone.0293963.s002.docx]

**Supplemental Table 1. Pearson correlation coefficients for age/gender, disease states, and HRQoL domains.**

|  | Gender^†^ | | Age | | HIV | | Diabetes | | Stroke | | HA | | HTN | | TB | | #C | | #C(I) | | #C(NI) | | Health | | Mobility | | Pain | | SC | | UA | | AD |
| --- | --- | --- | --- | --- | --- | --- | --- | --- | --- | --- | --- | --- | --- | --- | --- | --- | --- | --- | --- | --- | --- | --- | --- | --- | --- | --- | --- | --- | --- | --- | --- | --- | --- |
| Gender**^†^** | 1 | 0.15* | | 0.14* | | 0.10* | | 0.04* | | 0.03* | | 0.12* | | -0.07* | | 0.19* | | 0.11* | | 0.14* | | -0.10* | | -0.08* | | -0.11* | | -0.07* | | -0.09* | | -0.07* | |
| Age | 0.15* | 1 | | -0.005 | | 0.32* | | 0.11* | | 0.06* | | 0.54* | | 0.08* | | 0.47* | | 0.02^1^ | | 0.55* | | -0.47* | | -0.45* | | -0.48* | | -0.42* | | -0.47* | | -0.35* | |
| HIV | 0.14 | -0.005 | | 1 | | -0.08* | | 0.03* | | -0.009 | | -0.08* | | 0.05* | | 0.55* | | 0.96* | | -0.09* | | 0.05* | | 0.09* | | 0.06* | | 0.08* | | 0.07* | | 0.04* | |
| Diabetes | 0.10* | 0.32* | | -0.83* | | 1 | | 0.06* | | 0.05* | | 0.31* | | 0.001 | | 0.52* | | -0.08* | | 0.69* | | -0.18* | | -0.19* | | -0.20* | | -0.17* | | -0.20* | | -0.16* | |
| Stroke | 0.04* | 0.11* | | 0.03* | | 0.06* | | 1 | | 0.09* | | 0.11* | | 0.001 | | 0.29* | | 0.03* | | 0.34* | | -0.15* | | -0.13* | | -0.14* | | -0.12* | | -0.13* | | -0.13* | |
| HA^3^ | 0.03* | 0.06* | | -0.009 | | 0.05* | | 0.09* | | 1 | | 0.06* | | -0.001 | | 0.20* | | -0.008 | | 0.25* | | -0.07* | | -0.05* | | -0.09* | | -0.05* | | -0.06* | | -0.06* | |
| HTN^4^ | 0.12* | 0.54* | | -0.08* | | 0.31* | | 0.11* | | 0.06* | | 1 | | 0.01 | | 0.64* | | -0.07* | | 0.85* | | -0.32* | | -0.33* | | -0.32* | | -0.29* | | -0.34* | | -0.24* | |
| TB^5^ | -0.07* | 0.08* | | 0.05* | | 0.001 | | 0.001 | | -0.001 | | 0.01 | | 1 | | 0.23* | | 0.34* | | 0.008 | | -0.03* | | -0.02^2^ | | -0.03* | | -0.04* | | -0.03* | | -0.03* | |
| #C^6^ | 0.19* | 0.47* | | 0.55* | | 0.52* | | 0.29* | | 0.20* | | 0.64* | | 0.23* | | 1 | | 0.58* | | 0.77* | | -0.27* | | -0.23* | | -0.26* | | -0.21* | | -0.26* | | -0.20* | |
| #C(I)^7^ | 0.11* | 0.02^1^ | | 0.96* | | -0.08* | | 0.03* | | -0.008 | | -0.07* | | 0.34* | | 0.58* | | 1 | | -0.08* | | 0.03* | | 0.08* | | 0.05* | | 0.07* | | 0.06* | | 0.03* | |
| #C(NI)^8^ | 0.14* | 0.55* | | -0.09* | | 0.69* | | 0.34* | | 0.25* | | 0.85* | | 0.008 | | 0.77* | | -0.08* | | 1 | | -0.35* | | -0.34* | | -0.36* | | -0.31* | | -0.36* | | -0.27* | |
| Health^9^ | -0.10* | -0.47* | | 0.05* | | -0.18* | | -0.15* | | -0.07* | | -0.32* | | -0.03* | | -0.27* | | 0.03* | | -0.35* | | 1 | | 0.51* | | 0.58* | | 0.50* | | 0.55* | | 0.45* | |
| Mobility | -0.08* | -0.45* | | 0.09* | | -0.19* | | -0.13* | | -0.05* | | -0.33* | | -0.02* | | -0.23* | | 0.08* | | -0.34* | | 0.51* | | 1 | | 0.64* | | 0.73* | | 0.72* | | 0.58* | |
| Pain^10^ | -0.11* | -0.48* | | 0.06* | | -0.20* | | -0.14* | | -0.09* | | -0.32* | | -0.03* | | -0.26* | | 0.05* | | -0.36* | | 0.58* | | 0.64* | | 1 | | 0.60* | | 0.70* | | 0.61* | |
| SC^11^ | -0.07 | -0.42 | | 0.08* | | -0.17* | | -0.12* | | -0.05* | | -0.29* | | -0.04* | | -0.21* | | 0.07* | | -0.31* | | 0.50* | | 0.73* | | 0.60* | | 1 | | 0.75* | | 0.60* | |
| UA^12^ | -0.09* | -0.47* | | 0.07* | | -0.20* | | -0.13* | | -0.06* | | -0.34* | | -0.03* | | -0.26* | | 0.06* | | -0.36* | | 0.55* | | 0.72* | | 0.70* | | 0.75* | | 1 | | 0.61* | |
| AD^13^ | -0.07* | -0.35* | | 0.04* | | -0.16* | | -0.13* | | -0.06* | | -0.24* | | -0.03* | | -0.20* | | 0.03* | | -0.27* | | 0.45* | | 0.58* | | 0.61* | | 0.60* | | 0.61* | | 1 | |

**^†^**Gender is coded such that 0=man, 1=woman. Positive correlation coefficients indicate higher prevalence among women than men; negative correlation coefficients indicate higher prevalence among men than women.

*^1^p* < 0.05 *^2^p <* 0.01 **p* < 0.001

^3^Heart attack

^4^Hypertension, or high blood pressure.

^5^Active TB.

^6^Total number of conditions.

^7^Number of infectious conditions.

^8^Number of non-infectious conditions.

^9^Overall perceived health.

^10^Pain/discomfort.

^11^Self-care

^12^Usual activity.

^13^Anxiety/depression.
