# Supplementary material for: HIV, multimorbidity, and health-related quality of life in rural KwaZulu-Natal, South Africa: A population-based study
Source: PLoS One. 2024 Feb 21;19(2):e0293963. doi: 10.1371/journal.pone.0293963 (PMC10880982; doi:10.1371/journal.pone.0293963)
Supplement: S2 Table — (DOCX) [file pone.0293963.s003.docx]

**Supplemental Table 2. Variance inflation factors (VIF) for the first and second set of regression models.**

| **Predictors (Model 1)** | **VIF** |
| --- | --- |
| Intercept | N/A |
| Diabetes | 1.034 |
| History of stroke | 1.001 |
| History of heart attack | 1.003 |
| HIV | 1.03 |
| Hypertension | 1.035 |
| Active TB | 1.004 |
| **Predictors (Model 2)** |  |
| Intercept | N/A |
| Current smoker | 1.01 |
| Controlled diabetes | 1.02 |
| Uncontrolled diabetes | 1.04 |
| History of stroke | 1.01 |
| History of heart attack | 1.008 |
| Controlled HIV | 1.07 |
| On treatment, but uncontrolled HIV | 1.01 |
| Uncontrolled HIV | 1.04 |
| Hypertension, uncontrolled | 1.03 |
| Active TB | 1.04 |
| Controlled TB | 1.03 |

Note: These are the VIFs for each of the two models after controlling for age and gender. Any VIF above 1 indicates a modest degree of collinearity.
